# An exploratory study on functional connectivity after mild traumatic brain injury: Preserved global but altered local organization

**DOI:** 10.1002/brb3.2735

**Published:** 2022-08-22

**Authors:** Eunkyung Kim, Han Gil Seo, Min Yong Seong, Min‐Gu Kang, Heejae Kim, Min Yong Lee, Roh‐Eul Yoo, Inpyeong Hwang, Seung Hong Choi, Byung‐Mo Oh

**Affiliations:** ^1^ Department of Rehabilitation Medicine Seoul National University Hospital Seoul Korea; ^2^ Biomedical Research Institute Seoul National University Hospital Seoul Korea; ^3^ Department of Rehabilitation Medicine Seoul National University College of Medicine Seoul Korea; ^4^ Department of Radiology Seoul National University College of Medicine and Seoul National University Hospital Seoul Korea; ^5^ National Traffic Injury Rehabilitation Hospital Yangpyeong Korea

**Keywords:** concussion, functional connectivity, graph theory, resting state functional magnetic resonance imaging

## Abstract

**Introduction:**

This study aimed to investigate alterations in whole‐brain functional connectivity after a concussion using graph‐theory analysis from global and local perspectives and explore the association between changes in the functional network properties and cognitive performance.

**Methods:**

Individuals with mild traumatic brain injury (mTBI, *n* = 29) within a month after injury, and age‐ and sex‐matched healthy controls (*n* = 29) were included. Graph‐theory measures on functional connectivity assessed using resting state functional magnetic resonance imaging data were acquired from each participant. These included betweenness centrality, strength, clustering coefficient, local efficiency, and global efficiency. Multi‐domain cognitive functions were correlated with the graph‐theory measures.

**Results:**

In comparison to the controls, the mTBI group showed preserved network characteristics at a global level. However, in the local network, we observed decreased betweenness centrality, clustering coefficient, and local efficiency in several brain areas, including the fronto‐parietal attention network. Network strength at the local level showed mixed‐results in different areas. The betweenness centrality of the right parahippocampus showed a significant positive correlation with the cognitive scores of the verbal learning test only in the mTBI group.

**Conclusion:**

The intrinsic functional connectivity after mTBI is preserved globally, but is suboptimally organized locally in several areas. This possibly reflects the neurophysiological sequelae of a concussion. The present results may imply that the network property could be used as a potential indicator for clinical outcomes after mTBI.

## 1 INTRODUCTION

Traumatic brain injury (TBI) is a growing health concern worldwide with more than 69 million people experiencing new TBIs each year (Dewan et al., 2018). Structural and functional brain changes are caused even in mild TBI (mTBI), which is also known as a concussion (Levine et al., 2006). The brain changes are often accompanied by neurological symptoms and cognitive deficits, including memory loss, poor executive function, and inattention (McInnes et al., 2017). This leads to extensive research on mTBI, in particular, changes in functional brain network, since local brain changes result in alteration of the interactions between brain regions regardless of the anatomical distance (Cao & Slobounov, 2009). Particularly, there is substantial literature using resting‐state functional magnetic resonance imaging (rsfMRI) to investigate functional brain network changes in mTBI (Morelli et al., 2021). The results from these rsfMRI studies have indicated that individuals with mTBI have stronger functional connectivity than controls during the acute and subacute phases of injury (Churchill et al., 2017; Kaushal et al., 2019), although there was also a mixture of increased and decreased connectivity findings (Morelli et al., 2021). The increased connectivity was indicative of good memory, attention, and executive function, as well as clinically milder symptom severity in the mTBI group (Morelli et al., 2021). In contrast, increased connectivity within a month after injury was also reported to be associated with persistent post‐concussion symptom (PCS) (van der Horn et al., 2017).

Based on these findings, investigating topological characteristics of the brain can provide a new understanding of the complex nature of mTBI by quantitatively describing the altered characteristics of the brain in a mathematical framework (Bassett & Bullmore, 2009; Minati et al., 2013). From this point of view, graph theory has been used to describe functional brain networks based on a finite set of nodes and edges (Rubinov & Sporns, 2010). Previous studies assessing several graph‐theory measures representing network centrality, segregation, and efficiency have shown that mTBI changes the brain in suboptimal ways during the acute and subacute phases following mTBI (Boroda et al., 2021; Cao & Slobounov, 2009; Han et al., 2014; Hou et al., 2019; Messe et al., 2013; Rangaprakash et al., 2019; Shi et al., 2021; Spielberg et al., 2015; van der Horn et al., 2017; Zhou, 2017; ). Individuals with mTBI showed reduced degree and betweenness centrality in the frontal and occipital areas compared to controls (Cao & Slobounov, 2009; Zhou, 2017). The participation coefficient, which reflects the between‐module connectivity, was also reduced after mTBI in these areas (Han et al., 2014). Post‐concussive symptoms, anxiety, depression, and tau aggregation have been found to be correlated with brain network properties (Messe et al., 2013; van der Horn et al., 2017; Wooten et al., 2019; Zhou, 2017). The mTBI group with PCS (> 6 months) had a significantly reduced mean shortest path length globally compared to controls, but mTBI without PCS did not show such changes (Hou et al., 2019). There was a positive correlation between the performance of executive function and network efficiency of the dorsolateral superior frontal and anterior cingulate regions in young adults with mTBI (Shi et al., 2021).

Research on mTBI has been conducted on diverse populations, such as individuals with sports‐related mTBI and military‐based mTBI, as well as civilians. It has been known that there are different characteristics of injury between these populations (Asken et al., 2018), which might lead to different changes in the brain. To date, topological changes of the functional brain network during the acute and subacute phases of mTBI were investigated mostly in individuals who experienced blast‐ or sports‐related mTBI (Boroda et al., 2021; Cao & Slobounov, 2009; Han et al., 2014; Hou et al., 2019; Messe et al., 2013; Rangaprakash et al., 2019; Spielberg et al., 2015). In addition, there is preliminary evidence of the association between functional network properties and cognition after a single civilian mTBI unlike that of blast‐ or sports‐related mTBI (Caeyenberghs et al., 2017). Therefore, further studies are necessary to enrich the understanding of topological functional brain changes associated with cognitive function in civilian mTBI.

In the present study, the topological properties of intrinsic functional brain networks in civilian mTBI were examined using graph‐theory measures that represent network centrality, segregation, and efficiency. The cognitive performance of individuals with mTBI and that of the controls, who were matched for age and sex, were compared, including memory, executive function, and attention, and the relationship between network characteristics and cognitive performance after mTBI was investigated. We thoroughly explored the changes in the functional network, globally and locally. Due to the subtle nature of mTBI and previous findings that reported suboptimal organization of the brain after mTBI, we hypothesized that global network changes would be less significant than local changes. In addition, we speculated that altered organization of the functional brain network may be associated with impaired memory, executive function, and attention after mTBI.

## 2 MATERIALS AND METHODS

### 2.1 Participants

This study was approved by the local Institutional Review Board (1804‐047‐936). Twenty‐nine individuals with mTBI (14 men and 15 women, mean age 43.3 ± 14.5 years), and 29 control subjects (14 men and 15 women, mean age 42.8 ± 13.8 years) participated in this study. Glasgow Coma Scale scores of 13−15, loss of consciousness (<30 min), post‐traumatic amnesia (< 24 h), mental status change, or focal neurological deficits were defined as mTBIs (Head, 1993). All patients visited the concussion clinic within a month after injury and agreed to participate in the study. All patients underwent MR imaging within a month after injury (mean time since injury, 19.6 ± 6.5 days, range from 7 to 30 days), and individuals with a few micro‐bleeding lesions, nonspecific T2 high signal intensities, or negligible subdural or epidural hemorrhage were included in the study (see Figure S1 in Supporting Information). The control group included healthy participants without a history of TBI and a cognitive score at least 24, as assessed by the Korean‐Montreal Cognitive Assessment (K‐MoCA). The healthy participants should also have had no previous brain injury diagnoses or use of medication for psychiatric or neurological problems in the past 6 months. Control subjects were matched for age (stratification with 3‐year strata) and sex. In most cases, the clinical and cognitive tests were conducted on the same day of MR imaging, with a maximum interval of 1 week within the day of the scan. Written informed consent was obtained from the participants. Detailed demographical and clinical information of the participants is listed in Table 1.

**TABLE 1:** Demographic and clinical information of the patients

ID	Sex	Age	GCS	LOC	PTA	Time after injury	BDI	MoCA	FAB	GOSE	RPCSQ	EQ‐5D	Mode of injury	Identifiable neuropathology
01	F	23	N.A^a^	+^b^	+	30	12	27	17	6	21	7	Fell from bicycle	–
02	M	36	15	−^c^	−	29	30	22	17	5	39	10	Car accident	–
03	F	26	15	+	+	28	26	30	18	6	21	7	Fell from scooter	–
04	M	63	15	−	−	23	1	24	17	7	9	6	Fell from a height	A microbleed in the right cerebellum
05	F	39	15	−	−	12	21	27	17	5	43	10	Car accident	A few tiny clustered T2 high signal intensities in the left superior frontal gyrus
06	F	47	15	−	−	24	40	22	14	4	55	14	Car accident	–
07	F	52	N.A	−	+	18	27	24	17	5	49	8	Car accident	–
08	F	32	N.A	+	+	23	20	30	18	5	40	10	Pedestrian car accident	–
09	F	61	N.A	±^d^	+	18	1	28	17	7	4	5	Fell to the ground	–
10	M	45	N.A	+	±	17	25	25	16	5	41	9	Fell to the ground	–
11	F	35	N.A	+	+	23	11	29	17	6	32	8	Playing sports	–
12	M	47	15	−	+	14	7	25	17	5	12	7	Motorcycle accident	Mild T2 high signal intensity in the periventricular white matter. A microbleed in the right occipital lobe
13	M	58	N.A	+	+	12	10	26	16	6	28	8	Playing sports	–
14	F	48	15	N.A	−	11	13	28	16	6	20	6	Fell to the ground	–
15	F	30	15	−	−	22	21	25	16	5	42	10	Pedestrian car accident	−
16	M	24	N.A	+	−	27	12	26	16	5	13	7	Fell to the ground	−
17	F	57	N.A	+	−	26	19	22	14	6	31	8	Hit by an object	−
18	F	33	15	−	+	18	4	28	18	6	17	7	Fell to the ground	−
19	M	22	15	−	+	11	2	28	16	8	8	5	Playing sports	−
20	F	44	15	+	+	12	37	12	9	6	11	8	Pushed and hit by an object	−
21	M	27	15	+	−	24	6	28	18	6	23	8	Hit by an object	−
22	M	62	15	+	−	19	2	24	13	7	15	5	Fell to the ground	Numerous microbleeds in the brain. Chronic subdural hemorrhage in the right cerebral convexity
23	M	61	15	+	+	22	4	23	15	7	9	6	Fell to the ground	Linear shaped microbleed in the bilateral frontal and left temporal lobes
24	F	63	15	−	−	20	1	17	11	6	24	7	Hit by an object	−
25	M	74	15	+	+	22	10	26	17	7	14	6	Fell to the ground	−
26	M	36	15	±	−	21	13	26	18	7	24	8	Fell down the stairs	−
27	M	41	N.A	N.A	N.A	27	14	28	17	6	14	9	Fell to the ground	−
28	F	41	N.A	−	+	7	3	17	13	7	12	5	Fell to the ground	−
29	M	28	N.A	+	−	7	10	25	18	6	24	7	Fell to the ground	−

Abbreviations: BDI, Beck Depression Inventory; EQ‐5D, EuroQol‐5D; F, Female; FAB, Frontal assessment battery; GCS, Glasgow Coma Scale; GOSE, Extended Glasgow Outcome Scale; LOC, Loss of consciousness; M, Male; MoCA, Montreal Cognitive Assessment; PTA, Post‐traumatic amnesia; RPCSQ, Rivermead Post‐Concussion Symptom Questionnaire.

^a^ GCS, LOC, or PTA scores were unavailable (N.A.) in some cases due to absence of official reports or witness.

^b^ Presence (+) of LOC (<30 min) or PTA (<24 h).

^c^ Absence (−) of LOC or PTA.

^d^ Suspected (±) LOC or PTA.

### 2.2 Clinical and cognitive measures

All individuals completed the clinical evaluation consisting of the Beck Depression Inventory (BDI), K‐MoCA, and Frontal Assessment Battery (FAB). Individuals with mTBI were additionally assessed using the Extended Glasgow Outcome Scale, Rivermead Post‐Concussion Symptom Questionnaire, and EuroQol‐5D. Cognitive performance was assessed using the Computerized Neurocognitive Function Test (CNT40^®^; MaxMedica, Seoul, Korea), consisting of five different tasks: (1) the card‐sorting test, which assessed executive function by measuring the perseverative rate of set‐shifting, (2) the digit‐span test, which assessed working memory function by measuring forward and backward digit‐span size, (3) the verbal and (4) visual learning tests, which assessed verbal and visual memory function by measuring the number of words (or visual images) recalled in the first trial (A1), fifth trial (A5), after 20‐minute delayed trials (delayed recall), and those recalled from the first to fifth trials (A1–A5), and (5) the word color test evaluated selective and maintaining attention by measuring correct responses and the response time, when reading the word only, the color only, the color word, the word of the color word, and the color of the color word.

### 2.3 Image acquisition and preprocessing

Brain imaging data, including that of structural T1 and rs‐fMRI, were acquired using a 3‐T scanner (Magnetom Triotim; Siemens, Erlangen, Germany). The rs‐fMRI data underwent standard preprocessing using the FMRIB Software Library (FSL, version 6.0.1, http://www.fmrib.ox.ac.uk/fsl) (Smith et al., 2004). A detailed description of acquisition parameters and preprocessing steps are provided in the Supporting Information. In short, preprocessing steps included the following: discarding the first four volumes of rs‐fMRI data, motion and slice timing correction, normalization to standard Montreal Neurological Institute template space, spatial smoothing, motion‐related artifacts removal using ICA‐AROMA (Pruim et al., 2015), regressing out the signal from the white matter and cerebrospinal fluid, and bandpass filtering in the range of 0.01 < *f* < 0.1 Hz. We additionally estimated each subject's framewise displacement to quantify their movement (Supporting Information). Group comparison was performed by using the mean framewise displacement between the groups (Table 2). Spatial smoothing was applied to the data, although it may alter the structure of the functional brain networks, particularly those constructed by the regions of interest (ROIs)‐based approach (Alakörkkö et al., 2017). It can increase the signal‐to‐noise ratio and help identify motion‐related artifacts when applying ICA‐AROMA. Global signal regression was not applied, given concerns about bias to negative correlations (Murphy & Fox, 2017; Saad et al., 2012), which may lead to substantial alterations in graph‐theoretical measures.

**TABLE 2:** Group comparison between individuals with mild traumatic brain injury (mTBI) and healthy controls (HC) with regard to demographic information, clinical characteristics, head movement during scanning, and cognitive performance

	mTBI	HC	*p* value
Age (years)	43.3 ± 14.5	42.8 ± 13.8	0.90
Sex (women:men)	15:14	15:14	–
BDI	13.9 ± 10.9	3.9 ± 4.0	<0.00
K‐MoCA	24.9 ± 4.1	27.1 ± 2.0	0.01
FAB	16.0 ± 2.2	17.2 ± 1.2	0.01
Framewise displacement	0.09 ± 0.06	0.08 ± 0.03	0.56
Cognitive test* ^,^ ^†^			
Card sorting test (executive function)	48.4 ± 20.1	57.6 ± 14.6	0.03
Digit span test (working memory)			
Forward	48.2 ± 13.4	59.4 ± 16.4	0.00
Backward	49.8 ± 12.6	58.1 ± 12.2	0.01
Verbal learning test (auditory working memory)			
A1	49.5 ± 9.2	56.4 ± 9.1	0.00
A5	52.0 ± 15.0	67.4 ± 10.9	<0.00
Delayed recall	47.0 ± 16.8	59.6 ± 15.6	0.00
A1∼A5 (sum)	41.4 ± 9.8	54.1 ± 11.3	<0.00
Visual learning test (visual working memory)			
A1	57.5 ± 11.7	60.5 ± 7.8	0.12
A5	66.8 ± 8.3	68.8 ± 7.3	0.17
Delayed recall	64.6 ± 7.4	66.9 ± 7.9	0.14
A1∼A5 (sum)	58.6 ± 7.3	61.8 ± 7.6	0.06
Word color test (attention)			
Word (black)	48.1 ± 11.1	57.6 ± 11.1	0.00
Color only	40.7 ± 10.5	48.7 ± 12.4	0.01
Color word	41.2 ± 10.4	48.7 ± 10.9	0.01
Word of color word	42.4 ± 11.0	51.7 ± 11.5	0.00
Color of color word	39.4 ± 13.6	47.6 ± 13.6	0.01

Abbreviations: A1, recalled in the first trial; A1∼A5, recalled from the first to the fifth trials; A5, recalled in the fifth trial; BDI, Beck Depression Inventory; FAB, Frontal Assessment Battery; HC, healthy controls; K‐MoCA, Korean–Montreal Cognitive Assessment; mTBI, mild traumatic brain injury.

Data are the mean ± standard deviation.

* One participant with mTBI was excluded from the group comparison of the cognitive performance test because of poor compliance during the tasks.

^†^ Cognitive performance was compared between the groups using a one‐sided two‐sample *t*‐test.

### 2.4 Network construction

**FIGURE 1:**
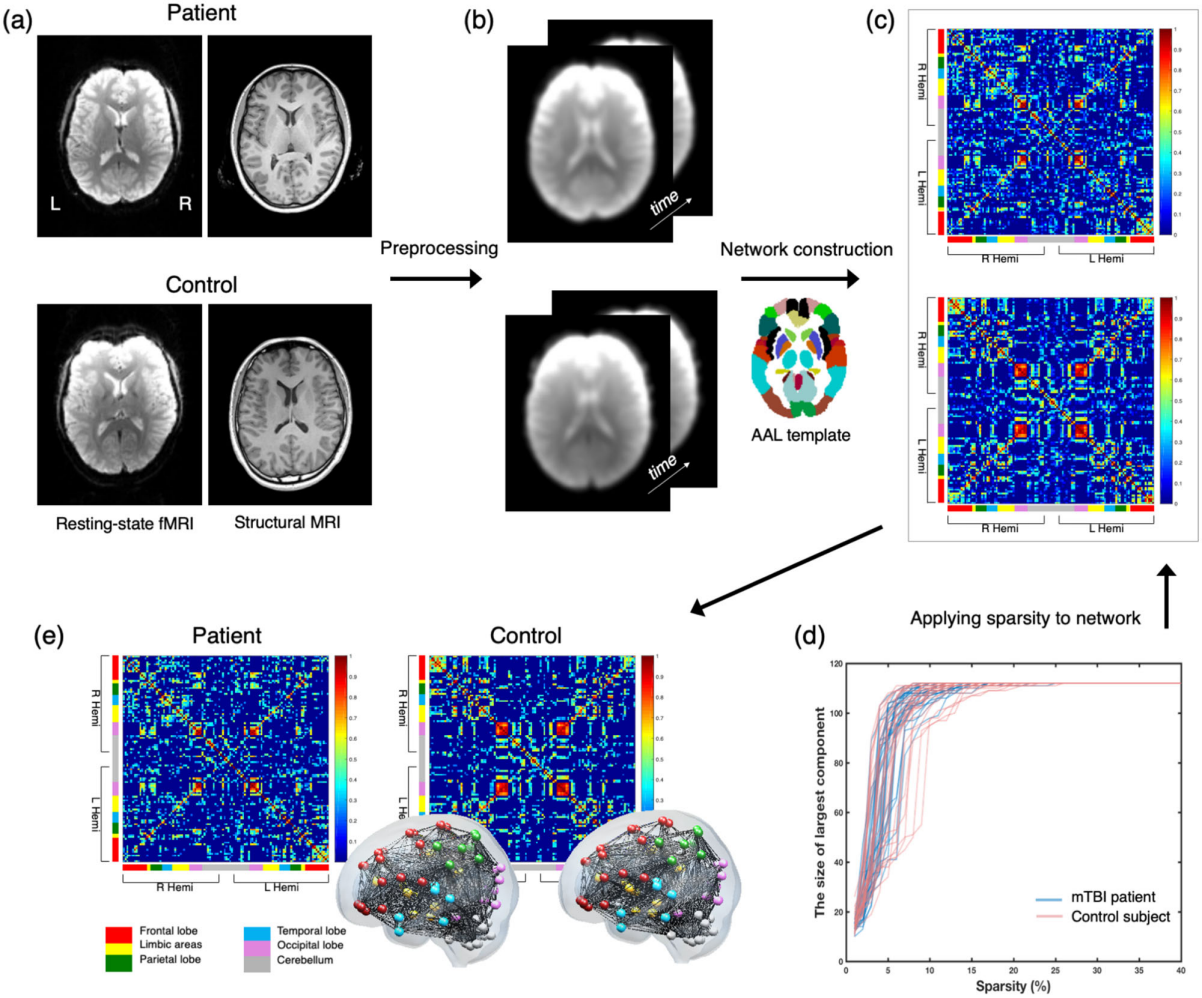
Framework of procedures for constructing functional connectivity. (a) Example of individual data from patient and control groups. (b) After the data was preprocessed, nodes were defined by a set of regions of interest from the automated anatomical labeling (AAL) template with time‐series data. (c) A positive correlation matrix across nodes after removal of age and sex from the data using the general linear model. The color of each axis indicates the region; red indicates the frontal lobe, yellow indicates limbic areas, green indicates the parietal lobe, light blue indicates the temporal lobe, pink indicates the occipital lobe, and gray indicates the cerebellum. The regions are ordered by right and left hemisphere (Hemi). (d) The sparsity strategy was applied to the connectivity, which thresholds the functional connectivity considered to control the sparsity of edges in the network. In this figure, the *x*‐axis indicates the sparsity of the network which is calculated using a simple equation: the real number of edges divided by the possible number of edges of the network. The *y*‐axis indicates the size of the largest connected components, ranging from 0 to 112. When the size of the largest connected component is maximized, that is, all nodes are connected, the cost of the network is minimized. Sparsity was set at 26.2 % in the present study. The blue lines indicate the mTBI patients while the red lines indicate the controls. (e) The functional connectivity matrix and brain image are displayed, which considers sparsity of edges.

Functional networks were constructed using MATLAB (The MathWorks Inc., MA, USA) and in‐house script. An overview of the functional connectivity analysis is given in Figure 1. A detailed description of node definition is provided in the Supporting Information. In short, a total of 112 nodes were used to construct the functional network based on the automated anatomical labeling template (see Table S1 in Supporting information). The edges were defined by the correlation coefficient between the averaged time‐series data of a pair of ROIs. A positive correlation matrix was constructed after age and sex were removed from the data using the general linear model, after removing negative correlation values (Figure 1c). Fisher *r*‐to‐*z* transformation was not applied in this study. Because arbitrarily selected thresholds lead to different numbers of edges between subjects (He et al., 2008), the positive correlation matrix was thresholded considering matrix sparsity (the number of edges divided by the possible number of edges) (Achard & Bullmore, 2007; Bassett et al., 2008). The sparsity of functional connectivity derived from each individual is visually presented in Figure 1d. The selected sparsity was 26.2% in this study, at which point the cost of the network was minimized and all nodes were connected at the same time (Figure 1e) as reported previously (Kim et al., 2014). Additionally, a sparsity range of 8−26% was also selected in steps of 1% to generate a functional network. The minimum sparsity among all the participants was 8.1%, at which point at least all the nodes of one participant were simultaneously connected.

### 2.5 Graph‐theory measures

The Brain Connectivity Toolbox (http://www.brain‐connectivity‐toolbox.net/) (Rubinov & Sporns, 2010) was used to estimate network properties. In the present study, network centrality was represented by betweenness centrality and strength of each node. Betweenness centrality highlights the importance of a node by estimating the number of shortest path‐length passes through a given node. Strength is the sum of connectivity strength of a given node with all other nodes. Network segregation was represented by clustering coefficient, calculated by averaging the intensity of all triangles, which are the sets of three nodes between a given node and its neighbors. A higher clustering coefficient indicates that a given node is tightly coupled with its neighbors, whereas a lower clustering coefficient indicates weak connections. Network efficiency was represented by local efficiency (Eloc) and global efficiency (Eglob) (Latora & Marchiori, 2001). Eloc estimates the average of the inverse shortest path length between the direct neighbors of a given node when it is removed. Eglob estimates the average of the inverse shortest path length among all pairs of nodes. Both are indicative of the capacity to transfer information within a local or global connectivity.

### 2.6 Statistical analysis

Clinical characteristics, including BDI, K‐MoCA, and FAB, were compared between the groups using a two‐sample *t*‐test (two‐sided test). Cognitive performance, which was represented by the *t*‐scores with a mean of 50 and standard deviation of 10, based on age‐matched normative samples, was compared between the groups using a two‐sample *t*‐test (one‐sided test).

A non‐parametric permutation test was performed to determine the significance of the difference between the graph‐theory measures in the two groups. To compare local network properties, for example, betweenness centrality of the node, *i*, was randomly assigned to the pseudo mTBI group and pseudo control group, and a two‐sample *t*‐test was performed. This randomization procedure was repeated 10,000 times to generate a null distribution of pseudo *t*‐values for node *i*. If the *t*‐value between the betweenness centrality of the node *i* in the mTBI and control groups was higher than the 97.5 percentile point of the null distribution, or lower than the 2.5 percentile point of the null distribution, the null hypothesis could be rejected. In this statistical analysis, multiple comparison correction was not conducted because of the small number of datasets relative to the large number of comparisons across nodes. The same procedure was repeated to analyze differences between the local network measures using the area under curve (AUC).

To compare global network property, that is, Eglob, this permutation test was performed at the global level, estimating the value per subject. In this comparison, if the *t*‐value between the Eglob of two groups was lower than 5 percentile points of the null distribution, the null hypothesis was rejected. To compare the betweenness centrality, strength, clustering coefficient, and Eloc from a global network perspective between the groups, the value of all nodes in each individual was averaged and compared using non‐parametric Wilcoxon's rank‐sum test (one‐sided test, *p* < 0.05) because the value was non‐normally distributed. Likewise, the global network properties were also compared using the AUC within the range of sparsity.

The relationship between graph‐theory measures in local network properties and cognitive performance in each group was examined by Pearson's correlation (*p* < 0.05). Multiple comparison correction across 16 CNT sub‐tests were conducted using false‐discovery rate correction (FDR) (Benjamini & Hochberg, 1995). If there was a significant correlation, the non‐parametric random permutation test was performed to determine that the relationship between cognitive performance and local network properties was significantly different between the groups. In detail, the local network properties of node *i* were randomly assigned to the pseudo mTBI group and the pseudo control group. After estimating the pseudo correlation coefficient, the value was transformed to the *z* value. *Z* comparison was performed repetitively 10,000 times to generate null distribution using the following equation.

$$\begin{array}{ccc} \mathrm{Zdif}f_{\mathrm{pseudo}} & = & \left(Z_{\mathrm{pseudo}-\mathrm{mTBI}}-Z_{\mathrm{pseudo}-\mathrm{control}}\right)/\mathrm{sqrt}\left(1/\left(N_{\mathrm{mTBI}}-3\right)\right.\\ & & \left.\pm \;1/\left(N_{\mathrm{control}}-3\right)\right) \end{array}$$

The original value of *z* comparison was compared to the null distribution to obtain statistical significance. Of 29 patients, one participant was excluded in the group comparison and correlation analyses with CNT subscores because of poor compliance during the task.

## 3 RESULTS

### 3.1 Comparison of clinical characteristics and cognitive performance

The mTBI group had significantly higher BDI scores (*p* < 0.001) and lower K‐MoCA and FAB scores (*p* < 0.05) compared to those of the controls (Table 2). Other than comparative scores of the visual learning test assessed visual memory function (A1, A5, delayed recall, and sum of the A1–A5 tests, *p* = 0.12, 0.17, 0.14, and 0.06, respectively) the mTBI group showed poorer performance in the executive function, working memory, verbal memory, and selective and maintaining attention (*p* < 0.05 across all tests, respectively, Table 2).

### 3.2 Global network characteristics

In the permutation test, the Eglob of the two groups was not significantly different (*p* > 0.05). The betweenness centrality, strength, clustering coefficient, and Eloc showed no difference between the groups from a global perspective (*p* > 0.05) (Table 3). The global network characteristics did not differ significantly within the range of sparsity (Table S2 in Supporting Information).

**TABLE 3:** Mean value of global network characteristics for individuals with mild traumatic brain injury (mTBI) and healthy controls (HC)

	mTBI	HC	*p* value
Global efficiency	0.28 ± 0.02	0.28 ± 0.02	0.16
Betweenness centrality	0.01 ± 0.00	0.01 ± 0.00	0.12
Strength	13.78 ± 1.19	14.21 ± 1.97	0.23
Clustering coefficient	0.29 ± 0.04	0.31 ± 0.07	0.20
Local efficiency	0.38 ± 0.04	0.39 ± 0.07	0.21

Abbreviations: HC, healthy controls; mTBI, mild traumatic brain injury.

*Notes*: Data are the mean ± standard deviation. Each nodal characteristic was averaged across all nodes. Thereafter, the average and standard deviation were estimated across all individuals in each group. Global network characteristics were compared between the groups using a one‐sided two‐sample *t*‐test.

### 3.3 Local network characteristics

**FIGURE 2:**
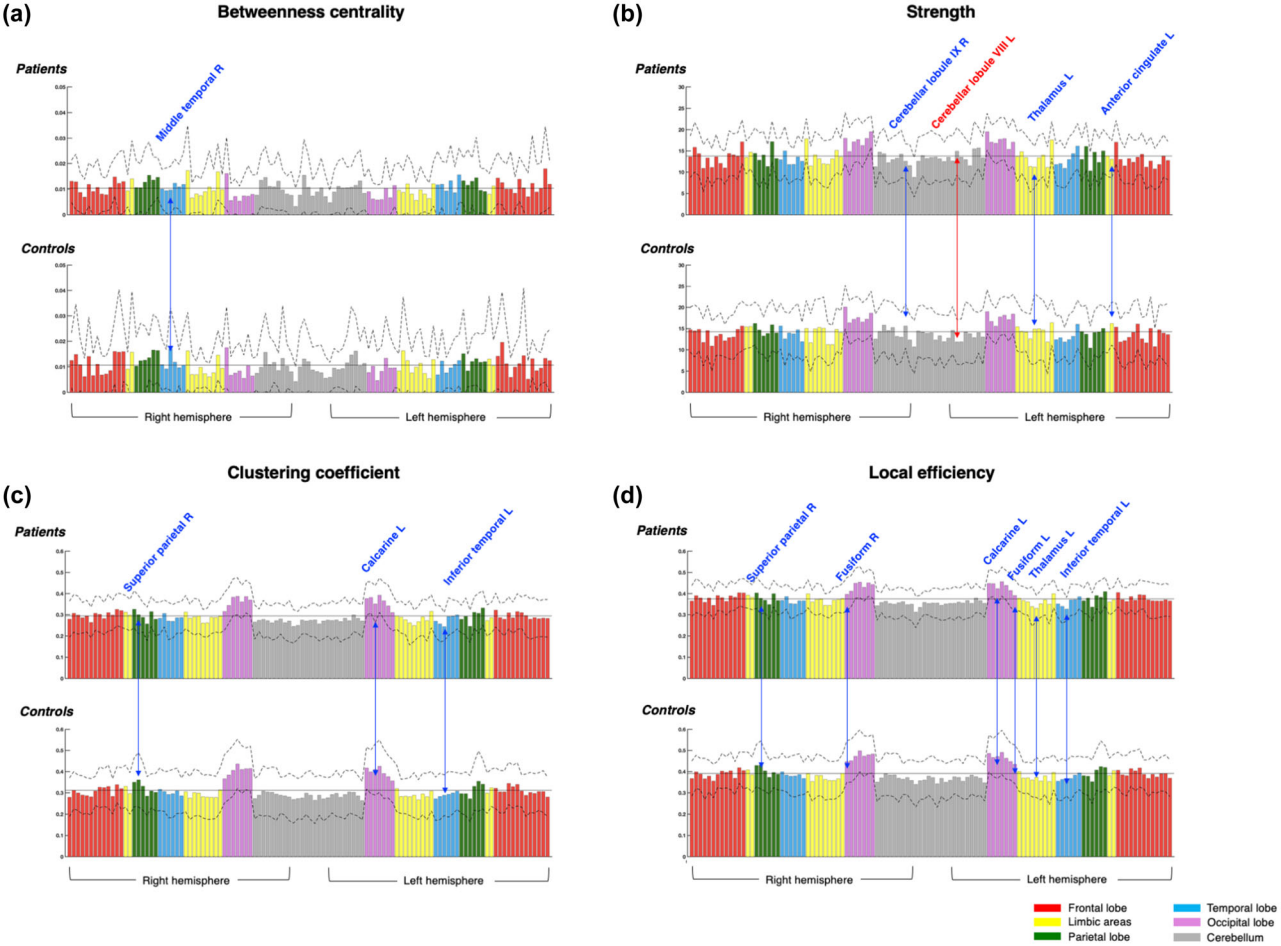
The results of the group comparison of the local network characteristics including (a) betweenness centrality, (b) strength, (c) clustering coefficient, and (D) local efficiency, which are displayed as a bar graph. The color of each bar was determined by the location of the brain as follows: red indicates the frontal lobe, yellow indicates limbic areas, green indicates the parietal lobe, light blue indicates the temporal lobe, pink indicates the occipital lobe, and gray indicates the cerebellum. The black solid line on the bar indicates the averaged value of all nodes over all individuals of each group while the black dashed line on the bar indicates one standard deviation above and below the average value of each node over all individuals of each group. The increased and decreased local network characteristics in the mild traumatic brain injury group compared to the controls are indicated with blue and red arrows, respectively. The regions showing significantly increased local network characteristics are listed on the bar graph in red while the significantly decreased local network characteristics are listed on the bar graph in blue. The significance of the group comparison was set at *p* < 0.05 in the non‐parametric random permutation test.

The significantly different local network characteristics are listed in Table 4. In terms of network centrality, the right middle temporal area showed decreased betweenness centrality in the mTBI group compared to that in the control group (Figure 2a). The right cerebellar lobule IX, left thalamus, and anterior cingulate areas showed decreased strength, while the left cerebellar lobule VIII showed increased strength in the mTBI group (Figure 2b). As for network segregation and efficiency, the left inferior temporal, right superior parietal, and left calcarine areas showed significantly decreased clustering coefficient and Eloc (Figure 2c,d). Decreased Eloc was also observed in the bilateral fusiform areas and the left thalamus.

**TABLE 4:** The significantly different network characteristics, including the betweenness centrality, strength, clustering coefficient, and local efficiency between the mild traumatic brain injury and control groups. The statistical significance was set at *p* < 0.05 in the random‐permutation test. The arrows pointing up and down indicated increased and decreased network characteristics in the patient group compared to the control group, respectively

Network characteristics	Region name	Lobe	↓	↑	*t*‐value
Betweenness centrality	Right middle temporal area	Temporal	↓		−2.10
Strength	Left anterior cingulate area	Limbic	↓		−2.56
	Left thalamus		↓		−2.06
	Right Cbll IX	Cerebellum	↓		−2.48
	Left Cbll VIII			↑	2.14
Clustering coefficient	Left inferior temporal area	Temporal	↓		−2.07
	Right superior parietal area	Parietal	↓		−2.11
	Left calcarine	Occipital	↓		−2.17
Local efficiency	Left inferior temporal area	Temporal	↓		−2.24
	Right superior parietal area	Parietal	↓		−2.10
	Left thalamus	Limbic	↓		−2.04
	Right fusiform	Occipital	↓		−2.17
	Left calcarine		↓		−2.05
	Left fusiform		↓		−2.00

Using AUC analysis within the range of sparsity, we observed that the right cerebellar lobule IX, left thalamus, and anterior cingulate areas showed decreased strength, while the right hippocampus showed increased strength in the mTBI group. The left calcarine showed reduced clustering coefficient and Eloc in the mTBI group (see Figure S2 and Table S3 in Supporting Information).

### 3.4 Association between local network characteristics and cognitive performance after mTBI

**FIGURE 3:**
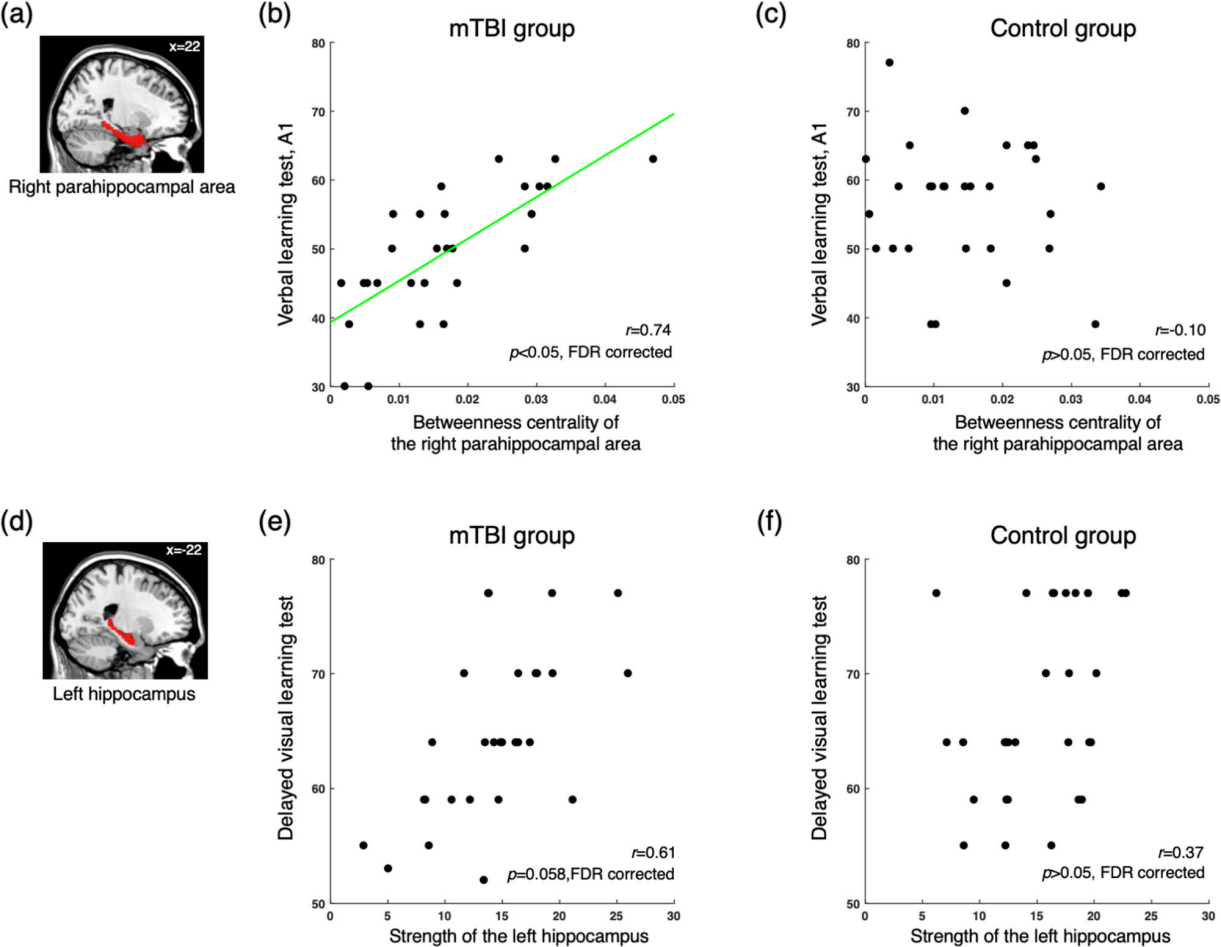
The relationship between the network characteristics and cognitive performance in the mild traumatic brain injury (mTBI) and control groups. (a) In the right parahippocampal area, (b) the betweenness centrality shows a significantly positive correlation with the scores of the verbal learning test A1 in the mTBI group (*r* = 0.74, *p* < 0.05, false‐discovery rate corrected), whereas (c) those in the control group show no association (*r* = −0.10, *p* > 0.05, false‐discovery rate corrected). (d) In the left hippocampus, (e) the strength shows a significantly positive correlation with the scores of the delayed visual learning test in the mTBI group (*r* = 0.61, *p* = 0.06, false‐discovery rate corrected), while (f) those in the control group show no association (*r* = 0.37, *p* > 0.05, false‐discovery rate corrected). Black dots indicate individuals within each group.

The betweenness centrality of the right parahippocampal area showed a significant positive correlation with cognitive performance assessed by verbal learning test A1 in the mTBI group (*r* = 0.74, *p* < 0.05 FDR corrected, Figure 3a), but not the control group (Figure 3b). The relationship was significantly different between the groups using the nonparametric random permutation test (*p* < 0.001). In the mTBI group, the score of word of color word test was positively correlated with the betweenness centrality of the cerebellar vermis IV–V (*r* = 0.66, *p* < 0.05 FDR corrected), but the correlation disappeared when the outlier subject was removed based on the median absolute deviation (Leys et al., 2013). Delayed recall in the visual learning test showed a marginal correlation with the strength of the left hippocampus in the mTBI group (*r* = 0.61, *p* = 0.06 FDR corrected, Figure 3c), but not the control group (Figure 3d). There was no such correlation in the control group.

## 4 DISCUSSION

The results of the present study demonstrate that topological characteristics of the functional brain network in the mTBI group did not differ significantly from those in the control group from a global perspective. Nevertheless, the results showed that local network characteristics in specific brain regions were altered in the mTBI group. The association between cognitive performance after mTBI and changes in network characteristics was also identified, suggesting a close relationship between local network changes and cognitive performance.

### 4.1 Preserved global network characteristics after mTBI

Individuals with mTBI showed network characteristics that were comparable to those of the control participants at a global level. This result seems to be plausible due to the subtle nature of mTBI. Nevertheless, this finding was inconsistent with those of previous studies that showed less efficient network communication in individuals with mTBI, especially in those with PCS. Participants with PCS in the mTBI group showed decreased network efficiency and centrality during the acute and subacute stages of injury while those without PCS did not compare to controls (Hou et al., 2019; Zhou, 2017). A longitudinal study over 3 months after injury is necessary to confirm whether the preserved global network characteristics after mTBI observed in the present study are related to the severity of PCS. Previous studies have reported comparable network properties at a global level between mTBI and control groups, with or without PCS (Messe et al., 2013; van der Horn et al., 2017). Such mixed results in global network changes across various studies may be due to the heterogeneous characteristics of mTBI and differences in the statistical analyses used.

### 4.2 Altered local network characteristics after mTBI

Decreased network centrality after mTBI was observed in the right middle temporal areas using betweenness centrality, and in the left thalamus and anterior cingulate area using strength. Each result represents reduced number of short cuts passing through the area and weakened connectivity strength of the given regions within the whole‐brain network. The decreased centrality may be related to impaired function after TBI. For example, individuals with TBI have impaired emotion recognition (Schmidt et al., 2010) and semantic information processing (McWilliams & Schmitter‐Edgecombe, 2008), both of which are activated in the right middle temporal area (Mitchell et al., 2003; St George et al., 1999). The decreased centrality may also be influenced by probable diffuse axonal injury observed in the left thalamus and the anterior cingulate area (Little et al., 2010; Wu et al., 2010). Additionally, there was spatial correspondence between the region showing reduced white matter integrity and the decreased local network characteristics in the mTBI group compared to the control group (Zhou, 2017).

We observed mixed results of changes in cerebellar areas, that is, increased strength of the left cerebellar lobule VIII, which is part of the skeleto‐motor divisions (Glickstein et al., 2011), and decreased strength of the right cerebellar lobule IX, which is part of the attentional or executive and default‐mode network‐related division (Guell & Schmahmann, 2020). This may explain the dissociated cerebellar functions in the motor and cognitive domains after mTBI.

It should be noted that increased clustering coefficient and Eloc were not seen in the mTBI group, suggesting that there was no particular brain region that was functionally more clustered or fault‐tolerant after mTBI during the subacute phase of injury. In contrast, decreased clustering coefficient and Eloc were observed mostly in the occipital areas. Double or blurred vision, deficits in saccadic eye movements, and photosensitivity are common vision disorders observed after TBI (Kapoor & Ciuffreda, 2002). There may be an association between impaired visual functions and decreased segregation of local network and efficiency of the occipital areas in the mTBI group. In addition, the left inferior temporal and the right superior parietal areas, which are part of the attention network, showed decreased clustering coefficient and Eloc in the mTBI group (Kim, 2015). These results are in line with those of a previous study that showed disrupted local network properties, particularly decreased efficiency in the functional network, including the attention network (Han et al., 2016). In addition, it has been reported that the weakened connections with neighbor nodes may lead to increased functional activity of the regions by means of compensatory activation (Kim et al., 2009).

### 4.3 Association of network characteristics and cognitive performance after mTBI

With the exception of the visual learning test, performances in the 12 subtasks included in CNT were significantly reduced in individuals with mTBI. Although cognitive performance before injury was unknown, these results indicate impaired cognitive performance due to mTBI.

Network centrality reflects that suboptimal changes of the brain after mTBI are associated with impaired cognitive performance. For example, significant positive correlation between the scores of the verbal learning test (A1) and betweenness centrality of the right parahippocampal area, which is activated during memory processing, was observed in the mTBI group (Eichenbaum et al., 2007). The marginal significance between the scores of the delayed visual learning test and the strength of the left hippocampus also supports this explanation. In a previous study, cognitive performance after TBI was correlated with the white matter integrity, suggesting a potential role of diffusion tensor measurement in clinical studies (Kim et al., 2019). Likewise, the present results suggest that local network characteristics may be particularly informative for predicting clinical outcomes in individuals with mTBI.

### 4.4 Limitations

First, although post‐injury cognitive ability was assessed in individuals with mTBI, premorbid ability could not be assessed in the present study. Information on the education level of the participants could be used as an indicator of premorbid cognitive function. Second, since mTBI is a heterogeneous brain injury, the small sample size in our study may affect the results. Individuals with mTBI with positive neuroimaging findings were also included in the present study. We additionally excluded five individuals with mTBI and observed that the main findings were not changed, namely preserved global but altered local organization of the functional brain network (see Table S4 in Supporting Information). In addition, although the permutation test was conducted, multiplicity of tests comparing all nodes with four local network properties was not corrected. Multiple comparison correction was eschewed as an exploratory investigation, which inevitably led to statistical inflation. Many previous studies similarly reported altered graph‐theory measures without correction for multiple comparison (Hou et al., 2019; Messe et al., 2013; Shi et al., 2021; Rowland et al., 2017). Nevertheless, this is a major concern and limitation of the study. Large sample data are needed to confirm the results in a statistically more conservative way. Further, it would be useful to include a control group matched by educational level and socioeconomic background in future research to reduce the possibility that group differences are caused by the normal variability among individuals prior to injury. Future studies are also warranted to unveil the effect of important symptoms after mTBI, such as depression or sleep problems, on brain network properties using subgroup analysis. Constructing a brain network by controlling the BDI scores was inappropriate, as it may remove the relevant brain signals contributed by the effect of a concussion. There is also a limitation of studies on functional connectivity based on the different methods of constructing the functional networks. This may affect the interpretation of this study's findings and generalizability. Specifically, the way of defining the node and edge in functional connectivity may change the results of group comparison (Hallquist & Hillary, 2018). In this study, we choose the AAL atlas instead of the recently developed fine‐grained functional atlases (Power et al., 2011; Schaefer et al., 2018) to define the node. It is because we want to maintain the signal‐to‐noise ratio as high as possible, and preclude a large number of statistical tests. In addition, we applied single‐density threshold and range‐of‐density threshold to threshold edge strength. This proportional thresholding could be problematic when clinical groups may have changes in the number and strength of functional connections (Hallquist & Hillary, 2018). Nevertheless, there was no significant difference in mean functional connectivity between the individuals with mTBI and the controls.

In this study, we primarily used single‐density threshold to construct the functional network. Although it can estimate graph measures from the same topological features in each individual, significant findings could have been missed since we did not consider the values across a reasonable range of network density. Thus, we analyzed the data again by considering a range of sparsity. The results showed that Eglob did not differ significantly, but reduced strength, clustering coefficient, and Eloc were still observed in areas similar those found in the analysis in which single density threshold was used.

## 5 CONCLUSION

The subtle nature of mTBI may have allowed for a globally preserved network, but locally, the brain was suboptimally organized in several areas. This possibly reflects the neurophysiological sequelae of a concussion. Our study contributes to the understanding of whole‐brain network changes after mTBI at global and local perspectives and may imply that the properties of the brain network could be used as a potential indicator for clinical outcomes after mTBI.

## CONFLICT OF INTEREST

The authors report no conflict of interest.

### PEER REVIEW

The peer review history for this article is available at https://publons.com/publon/10.1002/brb3.2735

## Supporting information


**Table S1**. Whole‐brain regions of interest as defined by the Automated Anatomical Label (AAL) atlas, sorted by lobes.
**Table S2**. Mean value of global network characteristics for individuals with mild traumatic brain injury (mTBI) and healthy controls (HC), which were estimated by using the positive correlation matrix thresholded by applying the range of sparsity threshold (8%−26%).
**Table S3**. The significantly different network characteristics, including the betweenness centrality, strength, clustering coefficient, and local efficiency between the mTBI and control groups. Statistical comparison was performed based on the AUC within the range of sparsity (8% −26%). The statistical significance was set at p < 0.05 in the random‐permutation test. The arrows up and down indicated increased and decreased network characteristics in the patient group compared to those of the control group, respectively.
**Table S4**. The significantly different network characteristics, including the betweenness centrality, strength, clustering coefficient, and local efficiency between the mTBI and control groups when excluding the individuals with presence of identifiable MRI lesion and their matching controls. The statistical significance was set at p < 0.05 in the random‐permutation test. The arrows up and down indicated increased and decreased network characteristics in the patient group compared to those of the control group, respectively.
**Figure S1**. Visualization of the identifiable pathologies on the brain in five individuals with mTBI. (a) A microbleed in the right cerebellum visualized using susceptibility weighted imaging (SWI). (b) A few tiny clustered T2 high signal intensities, which are shown using T2 flair image. (c) Mild T2 high signal intensity visualized in the periventricular white matter on T2 flair imaging. (d) A microbleed in the right occipital lobe, visualized on SWI. (e) Numerous microbleeds and (f) chronic subdural hemorrhage in the right cerebral convexity, which were visualized using SWI. (g) A linear‐shaped microbleed shown in the bilateral frontal and left temporal lobes using SWI.
**Figure S2**. The results of the group comparison of the local network characteristics including (a) betweenness centrality, (b) strength, (c) clustering coefficient, and (d) local efficiency. The bar graph represents the average of the estimated values of the AUCs across the range of sparsity (8‐26%) in each individual. The black solid line on the bar indicates the averaged value of all nodes over all individuals of each group while the black dashed line on the bar indicates one standard deviation above and below the average value of each node over all individuals of each group.

## Data Availability

The data that support the findings of this study are available on request from the corresponding author. The data are not publicly available due to privacy or ethical restrictions.
