# Supplementary material for: An exploratory study on functional connectivity after mild traumatic brain injury: Preserved global but altered local organization
Source: Brain Behav. 2022 Aug 22;12(9):e2735. doi: 10.1002/brb3.2735 (PMC9480924; doi:10.1002/brb3.2735)
Supplement: Supplementary file 1 — Table S1. Whole‐brain regions of interest as defined by the Automated Anatomical Label (AAL) atlas, sorted by lobes. Table S2. Mean value of global network characteristics for individuals with mild traumatic brain injury (mTBI) and healthy controls (HC), which were estimated by using the positive correlation matrix thresholded by applying the range of sparsity threshold (8%−26%). Table S3. The significantly different network characteristics, including the betweenness centrality, strength, clustering coefficient, and local efficiency between the mTBI and control groups. Statistical comparison was performed based on the AUC within the range of sparsity (8% −26%). The statistical significance was set at p < 0.05 in the random‐permutation test. The arrows up and down indicated increased and decreased network characteristics in the patient group compared to those of the control group, respectively. Table S4. The significantly different network characteristics, including the betweenness centrality, strength, clustering coefficient, and local efficiency between the mTBI and control groups when excluding the individuals with presence of identifiable MRI lesion and their matching controls. The statistical significance was set at p < 0.05 in the random‐permutation test. The arrows up and down indicated increased and decreased network characteristics in the patient group compared to those of the control group, respectively. Figure S1. Visualization of the identifiable pathologies on the brain in five individuals with mTBI. (a) A microbleed in the right cerebellum visualized using susceptibility weighted imaging (SWI). (b) A few tiny clustered T2 high signal intensities, which are shown using T2 flair image. (c) Mild T2 high signal intensity visualized in the periventricular white matter on T2 flair imaging. (d) A microbleed in the right occipital lobe, visualized on SWI. (e) Numerous microbleeds and (f) chronic subdural hemorrhage in the right cerebral convexity, which were vi [file BRB3-12-e2735-s001.docx]

**Supporting Information**

An exploratory study on functional connectivity after mild traumatic brain injury: preserved global- but altered local organization

Eunkyung Kim^a,b^, Han Gil Seo^a,c^, Min Yong Seong^a^, Min-Gu Kang^a,1^, Heejae Kim^a^, Min Yong Lee^a,2^, Roh-Eul Yoo^d^, Inpyeong Hwang^d^, Seung Hong Choi^d^, Byung-Mo Oh^a,c,e,f^

^a^Department of Rehabilitation Medicine, Seoul National University Hospital, Seoul, Korea

^b^Biomedical Research Institute, Seoul National University Hospital, Seoul, Korea

^c^Department of Rehabilitation Medicine, Seoul National University College of Medicine, Seoul, Korea

^d^Department of Radiology, Seoul National University College of Medicine and Seoul National University Hospital, Seoul, Korea

^e^National Traffic Injury Rehabilitation Hospital, Yangpyeong, Korea

^f^Institute on Aging, Seoul National University, Seoul, Korea

^1^Author Min-Gu Kang is currently affiliated with the Department of Physical Medicine and Rehabilitation, Dong-A University College of Medicine, Busan, Korea

^2^Author Min Yong Lee is currently affiliated with the Rehabilitation Medicine Center, Korea Workers’ Compensation and Welfare Service Incheon Hospital, Incheon, Korea

**Address correspondence to**

*Byung-Mo Oh, MD, PhD

Department of Rehabilitation Medicine, Seoul National University College of Medicine and Seoul National University Hospital, 103 Daehak-ro, Jongno-gu, Seoul, Korea, 03080

E-mail: [keepwiz@gmail.com](mailto:keepwiz@gmail.com)

**Materials and methods**

**Image acquisition and preprocessing**

Brain imaging data were acquired using a 3-T scanner (Magnetom Triotim; Siemens, Erlangen, Germany). Structural images were obtained using a sagittal 3D Turbo-FLASH sequence (image matrix 256 mm × 256 mm, voxel size 1 mm^3^, repetition time 1670 ms, echo time 1.89 ms, field of view 250 mm, flip angle 9°). rs-fMRI data were obtained using an interleaved slice acquisition with 116 volumes consisting of 35 consecutive images (image matrix 128 mm×128 mm, voxel size 1.9 mm×1.9 mm×3.5 mm, repetition time 3500 ms, echo time 30 ms, field of view 240 mm, flip angle 90°). Participants were instructed to stay awake with eyes closed and motionless without thinking of anything.

The rs-fMRI data underwent standard preprocessing using the FMRIB Software Library (FSL, version 6.0.1, <http://www.fmrib.ox.ac.uk/fsl)> (1). After discarding the first four volumes to stabilize the signal due to the T1 relaxation effect, the images were visually inspected to detect severe motion artifacts or brain lesions. Motion and slice timing were corrected using the FMRI Expert Analysis Tool, and the corrected data was normalized to the standard Montreal Neurological Institute (MNI) 152 template space using a two-stage registration process, including linear and nonlinear registrations. At first, individual functional data were linearly registered to the individual structural image using a rigid body transformation (Boundary-Based Registration). The registered functional data were normalized to MNI152 standard space using a transformation matrix constituted by applying the nonlinear transformation of the individual structural image to the MNI152 standard template. To increase the signal-to-noise ratio, the preprocessed image was spatially smoothed using a Gaussian kernel (sigma=2.55). Motion-related artifacts were identified and removed using ICA-AROMA (2), which automatically detects motion-related temporal and spatial components using independent component analysis. The signal from the white matter and cerebrospinal fluid was removed from the data and bandpass filtering was applied to retain the brain signal in the 0.01 < f < 0.1 Hz range. Because fMRI data are susceptible to contamination by micro-movements of the head, each subject’s framewise displacement (FD) was estimated to quantify their movement (3). The total proportion of outlier volumes greater than FD>0.5 mm was 0.5% for the mTBI group (17 out of 3,248 volumes) and 0.2 % for the control group (6 out of 3,248 volumes). Therefore, the outlier volumes were not removed from the data to avoid data loss in advanced post-processing steps.

**Node definition**

Functional networks were constructed using MATLAB (The MathWorks Inc., MA, USA) and in-house script. After data acquisition (Figure 1a) and preprocessing (Figure 1b), nodes were defined by a set of 90 cerebral and 26 cerebellar regions of interest (ROIs) based on the automated anatomical labeling template (see Table S1 in Supporting information). The bilateral olfactory and rectus areas were removed from the nodes due to potential signal susceptibility artifacts. The non-brain voxels, including the bilateral middle, superior, and medial orbitofrontal areas, were masked out to the further analyses by multiplication with thresholded smoothed data due to susceptibility effects. The time-series data of all voxels within a given ROI were averaged to represent the brain signal within the node.

**References**

1. Smith SM, Jenkinson M, Woolrich MW, Beckmann CF, Behrens TE, Johansen-Berg H, et al. Advances in functional and structural MR image analysis and implementation as FSL. Neuroimage. 2004;23 Suppl 1:S208-19.

2. Pruim RH, Mennes M, van Rooij D, Llera A, Buitelaar JK, Beckmann CF. ICA-AROMA: A robust ICA-based strategy for removing motion artifacts from fMRI data. Neuroimage. 2015;112:267-77.

3. Power JD, Barnes KA, Snyder AZ, Schlaggar BL, Petersen SE. Spurious but systematic correlations in functional connectivity MRI networks arise from subject motion. Neuroimage. 2012;59(3):2142-54.

| **Table S1.** Whole-brain regions of interest as defined by the Automated Anatomical Label (AAL) atlas, sorted by lobes. | | |
| --- | --- | --- |
| Lobe | ROI Number (R/L) | AAL Name |
| Frontal | 1/112 | Inferior frontal area, orbital part |
|  | 2/111 | Inferior frontal area, opercular part |
|  | 3/110 | Inferior frontal area, triangular part |
|  | 4/109 | Middle frontal area, orbital part |
|  | 5/108 | Middle frontal area |
|  | 6/107 | Superior frontal area, orbital part |
|  | 7/106 | Superior frontal area, medial orbital |
|  | 8/105 | Superior frontal area, medial |
|  | 9/104 | Superior frontal area, dorsolateral |
|  | 10/103 | Paracentral lobule |
|  | 11/102 | Supplementary motor area |
|  | 12/101 | Precentral area |
|  | 13/100 | Rolandic operculum |
| Limbic | 14/99 | Anterior cingulate and paracingulate areas |
|  | 15/98 | Median cingulate and paracingulate areas |
| Parietal | 16/97 | Postcentral area |
|  | 17/96 | Superior parietal area |
|  | 18/95 | Precuneus |
|  | 19/94 | Inferior parietal, but supramarginal and angular areas |
|  | 20/93 | Supramarginal area |
|  | 21/92 | Angular |
| Temporal | 22/91 | Superior temporal area |
|  | 23/90 | Heschl |
|  | 24/89 | Middle temporal area |
|  | 25/88 | Inferior temporal area |
|  | 26/87 | Temporal pole: superior temporal area |
|  | 27/86 | Temporal pole: middle temporal area |
|  | 28/85 | Insula |
| Limbic | 29/84 | Caudate nucleus |
|  | 30/83 | Lenticular nucleus, putamen |
|  | 31/82 | Lenticular nucleus, pallidum |
|  | 32/81 | Thalamus |
|  | 33/80 | Amygdala |
|  | 34/79 | Hippocampus |
|  | 35/78 | Parahippocampal area |
|  | 36/77 | Posterior cingulate area |
| Occipital | 37/76 | Fusiform |
|  | 38/75 | Inferior occipital area |
|  | 39/74 | Middle occipital area |
|  | 40/73 | Superior occipital area |
|  | 41/72 | Calcarine fissure and surrounding cortex |
|  | 42/71 | Cuneus |
|  | 43/70 | Lingual |
| Cerebellum | 44/69 | Cerebellum VIII |
|  | 45/68 | Cerebellum IV-V |
|  | 46/67 | Cerebellum VI |
|  | 47/66 | Cerebellum Crus I |
|  | 48/65 | Cerebellum Crus II |
|  | 49/64 | Cerebellum VIIb |
|  | 50/63 | Cerebellum VIII |
|  | 51/62 | Cerebellum IX |
|  | 52/61 | Cerebellum X |
|  | 53 | Vermis I-II |
|  | 54 | Vermis III |
|  | 55 | Vermis IV-V |
|  | 56 | Vermis VI |
|  | 57 | Vermis VII |
|  | 58 | Vermis VIII |
|  | 59 | Vermis IX |
|  | 60 | Vermis X |

| **Table S2**. Mean value of global network characteristics for individuals with mild traumatic brain injury (mTBI) and healthy controls (HC), estimated by using the positive correlation matrix thresholded by applying the range of sparsity threshold (8%–26%). | | | |
| --- | --- | --- | --- |
|  | mTBI | HC | P value |
| Global efficiency | 0.258±0.021 | 0.259±0.024 | 0.336 |
| Betweenness centrality | 0.014±0.004 | 0.015±0.003 | 0.390 |
| Strength | 10.267±2.390 | 10.513±2.496 | 0.272 |
| Clustering coefficient | 0.329±0.020 | 0.340±0.014 | 0.152 |
| Local efficiency | 0.417±0.020 | 0.427±0.017 | 0.288 |
| Abbreviations: mTBI, mild traumatic brain injury; HC, healthy controls. Data are the mean±standard deviation. Each nodal characteristic was averaged across all nodes and across all individuals in each group. Thereafter, the average and standard deviation were estimated across the range of sparsity in each group. Global network characteristics were compared between the groups using a one-sided two-sample t-test. | | | |

| **Table S3.** The significantly different network characteristics, including the betweenness centrality, strength, clustering coefficient, and local efficiency between the mild traumatic brain injury and control groups. Statistical comparison was performed based on the area under curve within the range of sparsity (8% -26%). The statistical significance was set at p<0.05 in the random-permutation test. The arrows up and down indicated increased and decreased network characteristics in the patient group compared to those of the control group, respectively. | | | | | |
| --- | --- | --- | --- | --- | --- |
| Network characteristics | Region name | Lobe | ↓ | ↑ | t-value |
| Strength | Right hippocampus | Limbic |  | ↑ | 2.13 |
|  | Left anterior cingulate area |  | ↓ |  | -2.36 |
|  | Left thalamus |  | ↓ |  | -2.39 |
|  | Right Cbll IX | Cerebellum | ↓ |  | -2.18 |
|  |  |  |  |  |  |
| Clustering coefficient | Left calcarine | Occipital | ↓ |  | -2.19 |
|  |  |  |  |  |  |
| Local efficiency | Left calcarine | Occipital | ↓ |  | -2.02 |
|  |  |  |  |  |  |

| **Table S4.** The significantly different network characteristics, including the betweenness centrality, strength, clustering coefficient, and local efficiency between the mild traumatic brain injury and control groups when excluding the individuals with presence of identifiable MRI lesion and their matching controls. The statistical significance was set at p<0.05 in the random-permutation test. The arrows up and down indicated increased and decreased network characteristics in the patient group compared to those of the control group, respectively. | | | | | |
| --- | --- | --- | --- | --- | --- |
| Network characteristics | Region name | Lobe | ↓ | ↑ | t-value |
| Betweenness centrality | Right precentral area | Frontal | ↓ |  | -2.37 |
|  | Right middle Temporal area | Temporal | ↓ |  | -2.37 |
|  | Right Cbll IV-V | Cerebellum |  | ↑ | 2.05 |
|  |  |  |  |  |  |
| Strength | Left medial superior frontal area | Frontal | ↓ |  | -2.06 |
|  | Left thalamus | Limbic | ↓ |  | -2.12 |
|  | Right Cbll IX | Cerebellum | ↓ |  | -2.09 |
|  |  |  |  |  |  |
| Clustering coefficient | Right superior parietal area | Parietal | ↓ |  | -2.25 |
|  | Right fusiform area | Occipital | ↓ |  | -2.22 |
|  | Right calcarine |  | ↓ |  | -2.73 |
|  | Right cerebellar crus I | Cerebellum | ↓ |  | -2.51 |
|  | Left inferior temporal area | Temporal | ↓ |  | -2.04 |
|  | Left calcarine | Occipital | ↓ |  | -2.74 |
|  | Left inferior occipital area |  | ↓ |  | -2.25 |
|  |  |  |  |  |  |
| Local efficiency | Right superior parietal area | Parietal | ↓ |  | -2.12 |
|  | Right fusiform area | Occipital | ↓ |  | -2.38 |
|  | Right calcarine |  | ↓ |  | -2.67 |
|  | Right cerebellar crus I | Cerebellum | ↓ |  | -2.30 |
|  | Left calcarine | Occipital | ↓ |  | -2.56 |
|  | Left inferior occipital area |  | ↓ |  | -2.47 |
|  | Left fusiform area |  | ↓ |  | -2.02 |


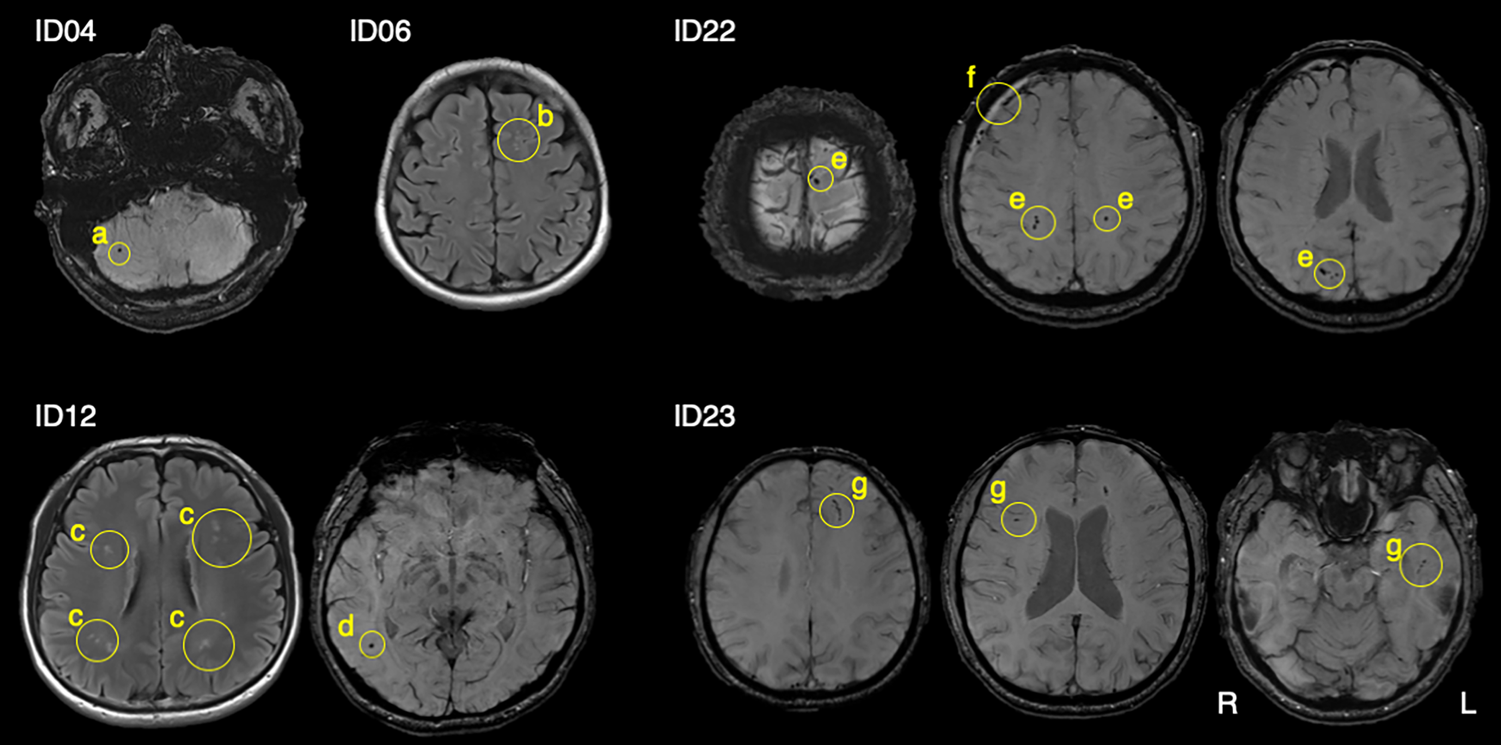


**Figure S1.** Visualization of the identifiable pathologies on the brain in five individuals with mTBI. (a) A microbleed in the right cerebellum visualized using susceptibility weighted imaging (SWI). (b) A few tiny clustered T2 high signal intensities, which are shown using T2 flair image. (c) Mild T2 high signal intensity visualized in the periventricular white matter on T2 flair imaging. (d) A microbleed in the right occipital lobe, visualized on SWI. (e) Numerous microbleeds and (f) chronic subdural hemorrhage in the right cerebral convexity, which were visualized using SWI. (g) A linear-shaped microbleed shown in the bilateral frontal and left temporal lobes using SWI.


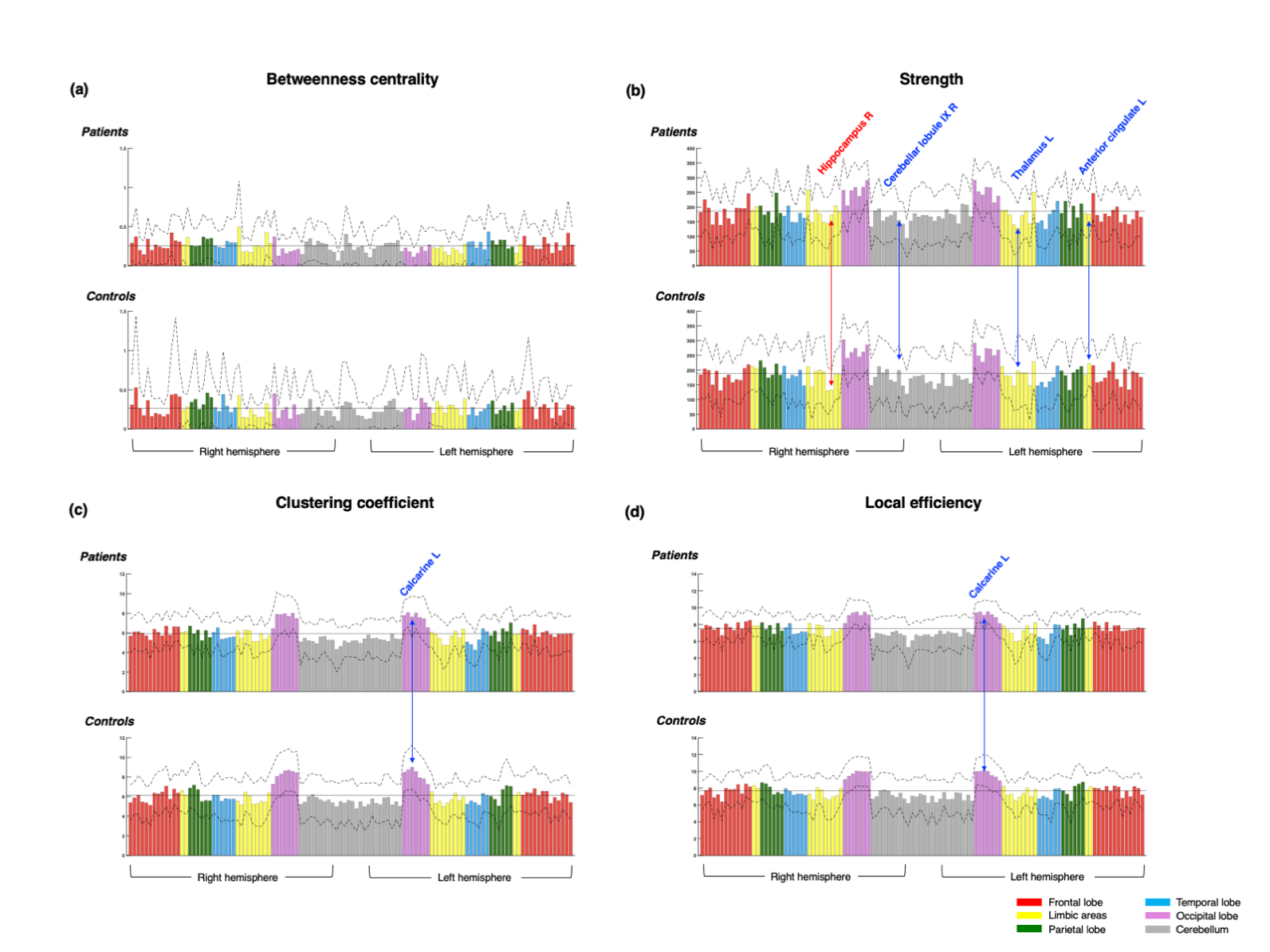


**Figure S2.** The results of the group comparison of the local network characteristics including (a) betweenness centrality, (b) strength, (c) clustering coefficient, and (d) local efficiency. The bar graph represents the average of the estimated values of the area under curves across the range of sparsity (8-26%) in each individual. The black solid line on the bar indicates the averaged value of all nodes over all individuals of each group while the black dashed line on the bar indicates one standard deviation above and below the average value of each node over all individuals of each group.
